# Contact Tracing During the COVID-19 Epidemic: Insights from the Experience of the Veneto Region in Italy

**DOI:** 10.3390/healthcare13030268

**Published:** 2025-01-30

**Authors:** Honoria Ocagli, Erica Marcolin, Filippo Da Re, Gloria Brigiari, Davide Gentili, Michele Mongillo, Michele Tonon, Federica Michieletto, Dario Gregori, Francesca Russo

**Affiliations:** 1 Unit of Biostatistics, Epidemiology and Public Health, Department of Cardio-Thoraco-Vascular Sciences and Public Health, University of Padova, 35131 Padova, Italy; honoria.ocagli@unipd.it (H.O.);; 2 Directorate of Prevention, Food Safety, and Veterinary Public Health-Veneto Region, 30123 Venice, Italy; davide.gentili@regione.veneto.it (D.G.); francesca.russo@regione.veneto.it (F.R.)

**Keywords:** contact tracing, COVID-19, hospitalizations, organizational models, pandemic control

## Abstract

Background: Nonpharmaceutical public health interventions, such as contact tracing (CT), have been widely implemented worldwide to mitigate the spread of coronavirus disease 2019 (COVID-19). Objectives: This study investigated the association between CT activity and COVID-19 cases, as well as the impact of timely contact with positive cases on hospitalizations in the Veneto region in northeastern Italy. Methods: Data sources included a CT-focused questionnaire, regional daily CT activity data, and a regional database of daily COVID-19 cases, hospitalizations, and intensive care unit (ICU) admissions. Negative binomial regression models were used to assess associations between CT activity and the number of positive cases, the number of hospitalizations, the time to contact cases, the number of positive cases traced, and the number of CT operators. Different organizational models (OMs) were compared in terms of their effectiveness. Results: Hospitalization rates decreased by 54% when index cases were contacted within 0–1 days compared with a five-day delay in the first period. During periods of increasing case numbers, hospitalizations decreased to 8% for contact ranges of 0–1 and 2–4 days. The increase in CT activity showed a 60% increase in daily activity per 100 cases in the third period, excluding external CT activities. Conclusions: These results emphasize the critical role of prompt and effective CT in controlling the spread of infectious diseases and reducing the burden on health care systems. Further research is warranted to explore the potential benefits and limitations of different organizational models in the context of contact tracing and public health management, as well as in a cross-cultural comparison.

## 1. Introduction

Nonpharmaceutical public health interventions (NPHIs) have been implemented throughout the world to reduce the spread of COVID-19, particularly during the early stages of the pandemic, when vaccine availability had not been fully implemented. A recent systematic review and meta-analysis by Iezadi et al. revealed that the application of NPHIs reduced the growth rate of cases of COVID-19 by 4.9% and decreased intensive care unit (ICU) admissions by 16.5% [1].

Among NPHIs, contact tracing (CT) has been recognized as an effective public health tool for controlling infectious diseases [2]. A recent systematic review evaluated the effectiveness of provider-initiated CT for transmissible infectious diseases, including COVID-19, tuberculosis, HIV, sexually transmitted infections (STIs), and measles [3]. Among the studies that evaluated provider-initiated CT, 29 (72.5%), including four of the six COVID-19 studies, reported that contact tracing interventions were associated with improvements in at least one outcome of interest. The review concluded that provider-initiated contact tracing may be an effective public health tool.

The literature emphasizes the importance of timely and thorough CT, which is often combined with other interventions, such as quarantine and isolation [1,3,4,5]. Eliminating delays in CT significantly reduced transmission rates [2,3] and decreased mortality growth rates by 4.8% and the reproduction number by 1.9 [1]. The early quarantine of exposed individuals prevented 44–81% of cases and 31–63% of deaths, while halving the reproductive number [6]. A review by Girum et al. [6] emphasized that quarantine, contact tracing, screening, and isolation are effective measures to prevent COVID-19, especially when they are implemented together. However, the literature highlights a lack of standardized implementation protocols and variations in CT systems across settings [3,7]. Modeling studies have identified highly effective CT policies, including manual CT with high coverage, hybrid manual and digital CT with high app adoption, secondary and bidirectional CT, and high-coverage CT in educational institutions. In Italy, digital CT was introduced through the mobile application ‘Immuni’ to complement traditional manual CT efforts. Despite its initial promise, Immuni’s adoption has remained limited, identifying only 44,880 COVID-19 cases (less than 1% of the total cases reported in Italy by the end of 2021) and generating 143,956 notifications during the same period [8]. While ‘Immuni’ has demonstrated some potential, its limited uptake and reliance on mandatory Green Pass certifications underscore significant challenges in integrating digital tools into broader CT systems [8,9].

CT can be classified as forward (identifying individuals to whom the disease may have spread) or backward (identifying the source of infection). The effectiveness of CT relies on the ability to reach the source of infection, making the development of strategic and efficient CT protocols essential [10].

This study aimed to evaluate the effectiveness of CT activity in the Veneto region. Specifically, the objectives were (i) to examine the relationship between the number of hospitalized individuals and the interval between the diagnosis and initiation of tracing activities, and (ii) to evaluate the impact of various organizational models in CT operations.

## 2. Materials and Methods

### 2.1. Data Sources

In this article, three data sources were considered: (i) the ’CT enhancement’ questionnaire (CCM), entitled “Enhancement by strengthening the role of Prevention Departments, particularly regarding conditions of increased susceptibility to the unfavorable consequences of infection (behavioral risk factors, chronic and multiple comorbidities, old age, etc.)”, (ii) the regional database of daily CT activity, and (iii) the regional COVID-19 database. The CCM questionnaire collected organizational data on CT activity across hospitals in the Veneto region between 13 December 2020 and 30 April 2022, with a focus on high-risk populations. The data were collected separately by local health units (LHUs). The variables collected included the daily number of new cases, daily CT activity, available personnel (physicians and other health care personnel dedicated to CT activity), daily cases contacted, cases at the end of the surveillance period, and cases contacted by different health care personnel. The second data source consisted of a regional daily monitoring system to evaluate the timeliness and completeness of interventions, as well as the workloads of the personnel involved. Data were collected for each LHU and covered the period from 1 January 2021 to 3 May 2022. The third dataset, covering 14 December 2020 to 14 February 2022, provided information on positive cases, including demographic data and hospitalization details, when available. These data sources are instrumental in understanding not only the dynamics of CT activity but also a broader approach to pandemic preparedness [11]. Specifically, the CCM questionnaire explicitly addresses organizational readiness and the identification of high-risk populations, which are key components of preparedness efforts in public health emergencies.

### 2.2. Definitions

A confirmed case of COVID-19 refers to any individual, regardless of symptoms, with a positive test for SARS-CoV-2 performed in a National Health Service (SSN) laboratory or a regional referral laboratory center [6].

Contact with a COVID-19 case is defined as any person exposed to a confirmed case within 48 h before or up to 14 days after symptom onset or any person exposed to an asymptomatic case within 48 h before the collection of a positive sample or up to 14 days after [11].

### 2.3. Contact Tracing Activity in the Veneto Region

Since the beginning of the pandemic in the Veneto region, containment measures, including testing and CT, have been implemented, as mandated by the D.G.R. n. 308 of 18 March 2021 [12]. During periods of low incidence and prevalence of SARS-CoV-2 infections, CT activity was intensified to detect and interrupt all transmission chains [12]. Public health authorities (SISP) supervised CT activity as described in the Istituto Superiore di Sanità (ISS) report on COVID-19 (n. 53/2020) [9]. The contact tracing process, as outlined in the Istituto Superiore di Sanità (ISS) report on COVID-19 (n. 53/2020) [11], involved three steps: First, the identification of contacts. This step focused on identifying potentially exposed people through interactions with confirmed cases. Second, contacts were traced. Once identified, contacts were followed up, including interviews and risk assessments. High-risk individuals were identified, and appropriate quarantine measures were recommended. Third, monitoring after contact was conducted. A 14-day monitoring period was initiated after contact. During this period, contacts were observed to detect symptoms, and their compliance with recommended containment measures was assessed [11].

### 2.4. Organizational Models

Different organizational models were considered when evaluating the number of positive subjects contacted and the number of CT operators. The same organizational model was applied in the Veneto region at the beginning of the pandemic. The number of personnel dedicated to CT increased during the pandemic, including those not directly employed by hospitals (externalized activity). In the second phase of the pandemic, from 30 October 2020, CT activities were further extended to general practitioners (MMGs), primary care pediatricians (PLSs), and emergency medical care (MCA) services. On the basis of the level of MMG/PLS involvement and the externalization of CT activity, the following organizational models (OM) were identified: (i) externalization of CT activity and high MMG involvement, (ii) externalization of CT activity and medium MMG involvement, (iii) no externalization of CT activity and medium MMG involvement, or (iv) no externalization of CT activity and low MMG involvement. The daily activity for each OM was proportional to the size of the population served by the respective organizational structures. The adoption of specific organizational models across regions underscores the importance of local flexibility in public health responses. Externalized contact tracing activities, for instance, provided regions with the capacity to scale operations during peaks in case numbers, while the varying involvement of GPs highlighted differences in the structural integration of healthcare systems at the regional level.

### 2.5. Data Analysis

This study covered the period from 1 January 2021 to 13 February 2022. A 7-day moving average was applied to the time series of COVID-19 cases and CT activity to mitigate the impact of erratic fluctuations, particularly during weekends. This methodological choice seeks to clarify the underlying trends by smoothing out inherent variations. The analysis adopted negative binomial regression models to evaluate the associations between daily activity and the number of positive subjects, between the number of hospitalizations and the range of days required to contact the case, and between the number of positive subjects contacted and the number of different operators performing CT.

Negative binomial regression models were chosen for their suitability for count data, offering advantages over alternatives such as Poisson regression. For example, negative binomial regression accommodates overdispersion, acknowledging and mitigating potential issues associated with variability. The results are presented as the incidence rate ratio (IRR) with a 95% confidence interval (CI) and corresponding *p* values, with the significance set at a *p* value lower than 0.05.

**Figure 1:**
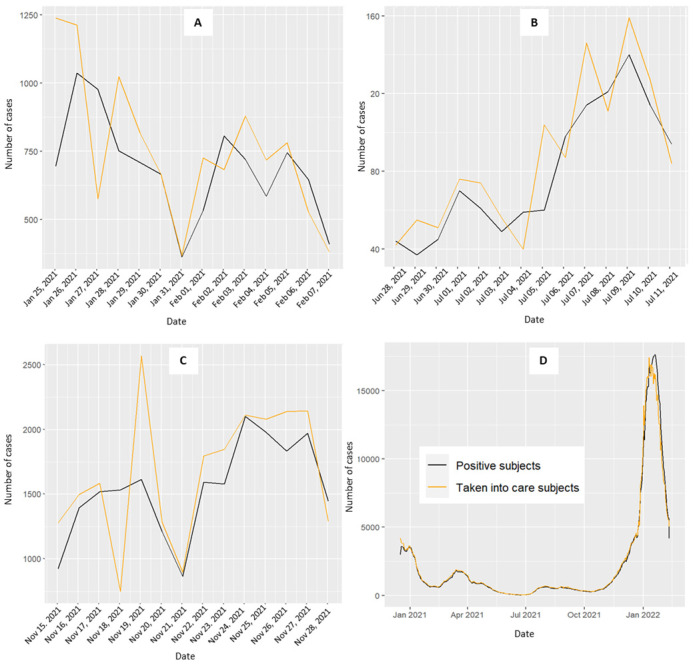
Daily trend of positive COVID-19 cases (black line) and the level of contact tracing activity (orange line) for the Veneto region (**D**). (**A**) decreasing curve not approaching zero from 25 January–8 February 2021; (**B**) stable curve at zero for 15 days from 28 June–12 July 2021; (**C**) increasing curve from 15–29 November 2021; (**D**) overall period.

To enhance granularity, the analysis incorporated distinct 15-day intervals. These periods were selected on the basis of the trends observed in the positive case curve: a decreasing curve not approaching zero (25 January–7 February 2021), a flattened curve near zero for 15 days (28 June–11 August 2021), and a rapidly increasing curve (27 December 2021–9 January 2022) (Figure 1). These intervals allowed for contextual relevance and enabled a more nuanced interpretation of our findings.

All analyses were performed with the statistical software R 4.2.0 [11].

## 3. Results

### 3.1. Associations Between Daily CT Activity and the Number of COVID-19-Positive Subjects

Figure 1 shows the trends of daily COVID-19-positive cases (black line) and CT activity (orange line) in the Veneto region. While the two curves generally mirror each other, a noticeable divergence occurs during the third period, emphasizing the dissimilarity compared with the previous two periods (Figure 1C). In January 2021, a rapid surge in COVID-19 cases was observed, with the CT daily activity curve following suit, albeit with a delayed response. The period from January to April 2021 showed an initial decline in the curves, coinciding with the implementation of a lockdown decreed on 14 January 2021 (DPCM), which ended on 5 March 2021. Between November and December 2021, the positive curve experienced rapid escalation, reaching its zenith in the early months of 2022. From May to November 2021, the daily counts remained near zero, with a slight increase between August and September 2021.

Figure S1 presents the daily CT activity during the three periods for each organizational model. As the dimensions of the areas with specific organizational models (OM) vary, the CT activity was proportionally aligned with the number of COVID-19 cases for each OM area to facilitate meaningful comparisons. Compared with the other models, the first organizational model exhibited a trend towards higher peaks in the CT activity. The second OM showed a trend with no significant increase or decrease compared with its counterparts. The third OM displayed lower levels of CT activity, whereas higher levels of activity were observed in the fourth model.

Table 1 reports the associations between the daily CT activity and the number of COVID-19 cases for the three periods considered in the Veneto region, categorized by the different organizational models. The second OM (externalization of CT activity and medium MMG involvement) served as the reference.

An increase in the daily activity of 60% (95% CI: 1.03–2.46, *p* value = 0.014) per 100 cases was observed in the first period for the third organizational model. During periods with a stable curve at zero (IRR = 2.56, 95% CI: 0.04–174, *p* value = 0.65 for the first model, IRR = 1.13, 95% CI: 0.04–32.7, *p* value = 0.94 for the third model, IRR = 1.29, 95% CI: 0.01–381, *p* value = 0.93 for the fourth model) and a rising curve, none of the OMs showed a significant increase in CT activity (IRR = 1.01, 95% CI: 0.87–1.16, *p* value = 0.94 for the first model, IRR = 1.11, 95% CI: 0.98–1.27, *p* value = 0.083 for the third model, IRR = 1.20, 95% CI: 0.98–1.47, *p* value = 0.082 for the fourth model) (Table 1).

### 3.2. Association Between the Number of Hospitalizations and the Range of Days Required to Contact the Patient

**Figure 2:**
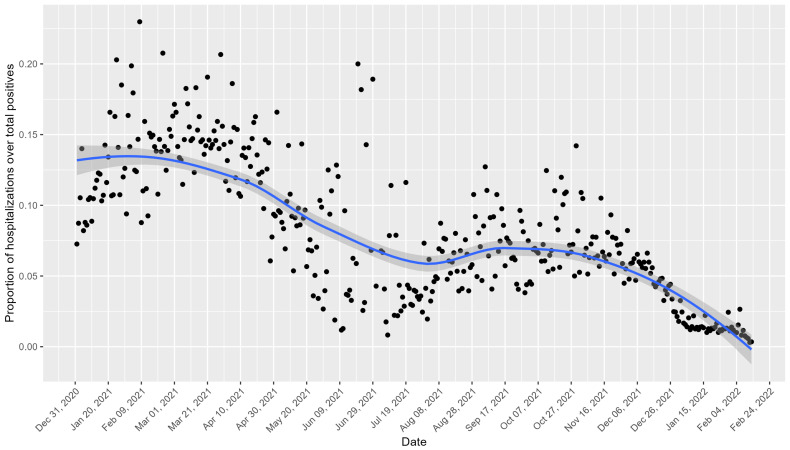
Daily trend of hospitalizations as a proportion of the total number of positive cases for the Veneto region. The black points represent the observed values, and the blue line indicates the smoothed trend with a confidence interval of 95%.

Figure 2 shows the daily trend of hospitalizations as a proportion of the total number of positive cases for the Veneto region. Peaks in the proportion of hospitalizations are notable in the early months of 2021 and October 2021, with declines observed during the summer months and around November 2021.

The observed declines in the hospitalization rates during early 2021 coincided with the initiation of Italy’s COVID-19 vaccination campaign, which began on 27 December 2020.

Figure S2 presents the daily hospitalization trend of the number of COVID-19 cases according to the four organizational models. The Health Prevention Department contacted COVID-19 patients within 0–1 days, 2–4 days, and five days after the diagnosis of positivity. Patients who were positive for COVID-19 were contacted within the same timeframes.

**Figure 3:**
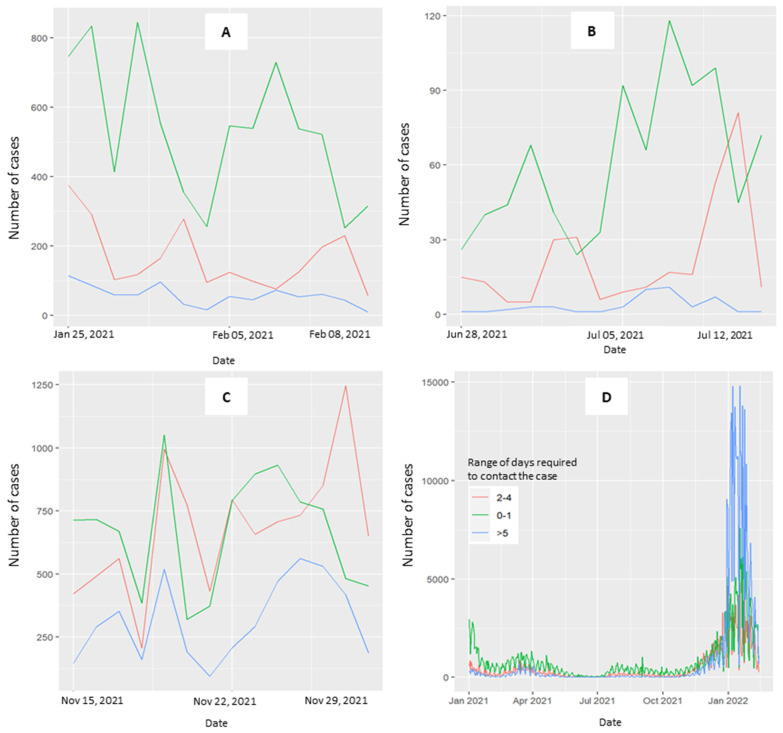
Trend of the range of days required to perform the CT activity. Patients testing positive for COVID-19 were contacted by the health prevention department at 0–1 days (green line), 2–4 days (red line), and 5 days (blue line) after the diagnosis of positivity. (**A**) Period with a decreasing curve, not approaching zero, from 25 January–8 February 2021. (**B**) Period where the number of cases with stable curves is at zero for 15 days, from 28 June–12 July 2021. (**C**) Period with a rising curve, from 15–29 November 2021. (**D**) Overall period.

Figure 3 explores the distribution of time required to contact cases across the three periods (3A, 3B, 3C) and throughout the overall study period (3D). When the curve is stable and curves toward zero, the range of 0–1 days is predominant. In the third period, the curves at 0–1 and 2–4 days converged. The curve for ≥5 days is consistently less represented across the three periods considered, with a notable exception occurring in January 2022 (Figure 3D, overall period).

Table 2 shows a 54% decrease in hospitalizations (95% CI: 0.27–0.79, *p* value = 0.003) when the index cases were contacted within 0–1 days compared with those contacted after five days in the first period.

In the rising curve period, the decrease in hospitalization was limited to 8% for the 0–1 day range (95% CI 0.83–1.02, *p* value = 0.11) and the 2–4 day range (95% CI 0.83–1.01, *p* value = 0.11) (Table 3). Notably, there was a 7% increase in the CT activity during the third period across all regions and a substantial 32% increase in model 4 compared to model 2 (95% CI 1.02, 1.71; *p* value = 0.037).

## 4. Discussion

The results of this study provide valuable insights into the association between CT activity and the number of COVID-19-positive cases, as well as the nuanced interplay between the number of hospitalizations and the timing of the contact with patients testing positive in the Veneto region.

CT activity, a pivotal tool for interrupting SARS-CoV-2 transmission, has proven particularly effective in mitigating the COVID-19 pandemic when implemented promptly. This impact was particularly pronounced in the early phases, as exemplified by the experience in Belgium [13].

The main results of this study are noteworthy: a significant reduction in hospitalizations occurred when CT was performed within 0–1 days of a positive result. The decline in hospitalization rates observed in early 2021 likely reflects the combined effects of multiple interventions, including the initiation of the COVID-19 vaccination campaign. The vaccination efforts, which were prioritized for vulnerable populations, may have substantially reduced severe cases, contributing to the observed trends. The organizational models, even in diverse settings, displayed a similar range of days during stable periods, with zero cases for fifteen days. In particular, a model that did not include externalized CT activity demonstrated an improved performance during rapid surges in the number of positive cases.

The Italian experience with CT during the COVID-19 pandemic revealed significant regional differences, influenced by the organizational strategies, testing capacity, and the impact on hospitalizations. For example, in Friuli Venezia Giulia (FVG), a cluster in Remanzacco (6000 inhabitants) was successfully managed through prompt CT, with 143 contacts traced. Of these, 54 were quarantined, and swabs collected from close contacts identified 18 confirmed cases, 61% of whom were asymptomatic. Only one patient required hospitalization, underscoring the role of early detection in preventing severe outcomes and reducing the hospital burden [14]. In the Province of Trento, during Phase I (March–April 2020), 6690 contacts were traced from 2812 cases, yielding a secondary attack rate of 13.3%. Most contacts (56%) were household members, whereas workplace exposures carried higher risks (15.8%). Delays during peak caseloads, with an average of 3.8 days between the last contact and follow-up initiation, may have contributed to severe cases and hospitalizations. Cohabitant exposure remained critical, contributing to 14.1% of secondary cases [15]. Piedmont initially prioritized symptomatic cases and was slower to expand its testing capacity. Nasal swabs ordered by general practitioners began only in May 2020, nearly three months later than in Veneto. This delay resulted in longer times from symptom onset to isolation, likely contributing to higher hospitalization rates and increased transmission rates [16].

In our experience, a shorter range of days required to initiate the CT activity correlated with a substantial decrease in the number of hospitalizations. There were 42% (95% CI: 0.48–0.69, *p* value < 0.001) and 24% (95% CI: 0.65–0.89, *p* value < 0.001) reductions, respectively, for the periods ranging from 0–1 and 2–4 days compared with the periods exceeding five days. This holds across the three periods analyzed, where the range of 0–1 days is prevalent and consistent with the observed reduction in hospitalization rates. These findings align with other findings reported in the literature [17]. Similarly, Jeon et al. [18,19] reported that the average number of hospitalizations was 0.1% to 1.5% when CT was effectively sustained over 60 days. The timing between the index case’s infection and the secondary infections, estimated at 4–5 days [20], aligns with the recommendations of Juneau et al. [4], emphasizing the critical window of 2–3 days from symptom onset to isolate cases and quarantine contacts [4]. This underscores the urgency of the prompt initiation of CT, particularly in symptomatic index cases. The brief window for implementing preventive measures is crucial, considering that infected individuals may not always exhibit symptoms, necessitating swift intervention [21,22].

However, this study also revealed that the effectiveness of contact tracing in reducing hospitalizations diminishes during periods of increasing cases. This reflects the challenges CT systems face in rapidly scaling up during surges [23]. This underscores the imperative for surge capacity and proactive planning to manage increased workloads during outbreaks.

Since the onset of the pandemic, the organizational model for contact tracing (CT) activity in the Veneto region has remained constant. On 30 October 2020, the CT strategy in the region strategically involved general practitioners (GPs) in the dual roles of testing and identifying index cases. These comprehensive CT teams were made up of qualified professionals, including public health doctors, physicians, health care assistants, and, at times, non-healthcare workers, all of whom were equipped with specialized training. This strategic approach responded directly to the increasing case numbers, adhering to recommendations from the European Centers for Disease Prevention and Control (ECDC) [24]. In adopting a standardized approach, the Veneto region opted to use a common platform to collect data related to epidemiological interviews. Data were collected directly during the interview process and seamlessly shared among the public health departments of all local health units and GPs, including the MMGs, PLSs, and MCAs. A comparative analysis revealed that a model involving GPs without externalization of the CT activity outperformed its counterpart in all three periods considered. In particular, the increase in CT activity was substantial—1.60 (95% CI (1.03–2.46, *p* value = 0.014), 1.13 (95% CI 0.04–32.7, *p* value = 0.94), and 1.11 (95% CI 0.98–1.27, *p* value = 0.083)—in the period with a decrease in cases not approaching zero, in the period with zero cases, and during a rising curve, respectively. During a rapid increase in cases, the fourth organizational model (no externalization of CT activity and low MMG involvement) demonstrated a remarkable 20% increase in activity (95% CI 0.98–47, *p* value = 0.082). This strategic evolution underscores the adaptability and effectiveness of involving GPs in the CT process, particularly during dynamic situations. The nuanced approach to externalization and GP involvement has proven crucial in navigating various scenarios, from periods of decline to those marked by a rapid surge in cases.

The involvement of personnel lacking a public health background, as suggested by the ECDC, requires significant coordination efforts [24]. In instances where externalization is not planned, in-house staff, already trained, can be swiftly activated. The performance superiority of the fourth model over the second model during a rapid increase may be attributed to its reduced dimension (658,279 people vs. 1,543,434 and 1,772,670 for the third and second models, respectively). Particularly for the fourth model, this agility in adaptation may be linked to its more limited pool of personnel, allowing for more nimble adjustments compared with larger units dealing with greater population demands and potentially facing more significant challenges. The choice of organizational models by different regions during the pandemic appeared to be influenced by several factors, including the availability of human resources, the level of epidemiological pressure, and the existing healthcare infrastructure. For example, regions with greater resource constraints may have relied more heavily on externalized contact tracing to handle surges in cases, while those with a robust integration of GPs into public health strategies opted for non-externalized models. These decisions reflect the need for adaptability in the face of rapidly changing circumstances and resource availability.

The observed reduction in COVID-19 cases during lockdown periods echoes findings from previous studies, highlighting the effectiveness of combining lockdowns and other nonpharmaceutical interventions (NPIs) to control COVID-19 transmission rates [25].

### Limitations

It is imperative to interpret these results in light of several limitations. The four organizational models employed different sample collection methods, using both PCR and antigenic tests in varying proportions. This variance may introduce a potential bias related to the time of test reporting, particularly in the organizational models that predominantly utilized PCR samples.

Furthermore, the difference in dimensions among the organizational models may influence their ability to cope with an increasing number of cases. We normalized the activity level to the number of cases in each organizational model to address this disproportion.

Additionally, the degree of externalization can be correlated with the dimensions of the organizational model, potentially influencing the performance outcomes.

A key limitation of this study is the potential influence of concurrent public health interventions, such as vaccination campaigns, lockdowns, and curfews, which were implemented during the same period as the contact tracing activities. While our analysis highlights the association between prompt CT and a reduced rate of hospitalizations, it is not possible to fully disentangle the effects of CT from these other measures.

## 5. Conclusions

These findings collectively underscore the critical role of swift CT in mitigating the spread of COVID-19, particularly in the face of increasing case numbers. The study’s nuanced exploration of the interplay between CT activity, case identification, and subsequent hospitalizations provides crucial insights for public health decision-makers, allowing them to manage and mitigate the impact of the ongoing pandemic. Nevertheless, optimizing CT strategies demands further research and meticulous consideration of diverse organizational models.

Since the onset of the COVID-19 pandemic, the Veneto region exemplified a rapid and effective response, as evidenced by the experience of the Vò municipality [26]. The backbone of this robust response lies in the organization of the CT activities, leveraging a resilient public health department, the consistency of data collection, and the standardization of procedures in different local health units. This strategic approach highlights the region’s proactive stance and emphasizes the importance of cohesive and standardized practices in pandemic control efforts. To further enhance CT strategies in future crises, scaling CT resources during early phases is crucial to ensure timely activation, within 0–1 days, which significantly reduces hospitalizations. Additionally, surge capacity planning must prioritize flexibility, integrating general practitioners (GPs) and leveraging in-house trained personnel for better adaptability during case surges.

The analysis of organizational models (OMs) in the Veneto region identifies key strategies for improving CT effectiveness. Models involving GPs and pediatricians (MMG/PLS) demonstrated greater efficiency during surges by leveraging local networks for early case identification. Non-externalized models (e.g., OM4) presented a 20% increase in CT activity during rapid surges, reflecting the agility of smaller, well-coordinated teams. Importantly, timeliness remained critical: contact with patients within 0–1 days was consistently associated with a significant reduction in hospitalizations.

From a broader perspective, the Veneto model highlights the importance of combining standardized data collection, coordinated public health departments, and the integration of local health care professionals to increase system resilience. The adoption of digital platforms for real-time monitoring of CT activity further strengthens workflows, prioritizes high-risk contacts, and supports evidence-based decision-making.

To ensure preparedness for future emergencies, public health systems must prioritize scalable CT resources, real-time digital tools, and surge capacity planning. These measures enhance the system agility, enabling timely responses and the effective containment of emerging threats.

## Figures and Tables

**Table 1:** A negative binomial regression model was used to evaluate the association between daily activity and the number of patients testing positive for COVID-19. The results are reported for the Veneto region according to the different organizational models (OM) for the three periods considered. The incidence rate ratio (IRR) with a 95% confidence interval (CI) and the *p* value are reported for each period.

	25 January–8 February 2021		28 June–12 July 2021		15–29 November 2021	
	Decreasing Curve Not Approaching Zero		Stable Curve at Zero for 15 Days		Rising Curve	
	IRR (95% CI)	*p* Value	IRR (95% CI)	*p* Value	IRR (95% CI)	*p* Value
Veneto region	1.21 (1.10–1.33)	<0.001	2.35 (1.42–3.95)	<0.001	1.06 (1.04–1.08)	<0.001
Mod1 vs. Mod2	1.11 (0.63–1.97)	0.72	2.56 (0.04–174)	0.65	1.01 (0.87–1.16)	0.94
Mod3 vs. Mod2	1.60 (1.03–2.46)	0.01	1.13 (0.04–32.7)	0.94	1.11 (0.98–1.27)	0.08
Mod4 vs. Mod2	1.38 (0.71–2.68)	0.33	1.29 (0.01–381)	0.93	1.20 (0.98–1.47)	0.08

Mod1 = first organizational model, Mod2 = second organizational model, Mod3 = third organizational model, Mod4 = fourth organizational model, IRR = incidence rate ratio, CI = confidence interval.

**Table 2:** A negative binomial regression model was used to study the association between the number of hospitalizations and the range of days required to contact the case at a regional level. The incidence rate ratio (IRR) with its 95% confidence interval (CI) and the *p* value are reported.

25 January–8 February 2021				28 June–12 July 2021				15–29 November 2021			
Decreasing Curve Not Approaching Zero				Stable Curve at Zero for 15 Days				Rising Curve			
Range of Days	IRR	95% CI	*p* Value	Range of Days	IRR	95% CI	*p* Value	Range of Days	IRR	95% CI	*p* Value
2–4 vs. ≥5	0.44	0.25, 0.76	0.002	2–4 vs. ≥5	1.02	0.14, 5.80	≥0.9	2–4 vs. ≥5	0.92	0.83, 1.01	0.08
0–1 vs. ≥5	0.46	0.27, 0.79	0.003	0–1 vs. ≥5 *	0.84	0.84, 4.77	0.9	0–1 vs. ≥5	0.92	0.83, 1.02	0.11

IRR = incidence rate ratio, CI = confidence interval, * the increase is related to ten subjects and not an increase of 100 subjects as in the other estimates.

**Table 3:** The effectiveness of the contact tracing activity considering the number of positive cases and those contacted by CT.

	25 January–8 February 2021			28 June–12 July 2021			15–29 November 2021		
	Decreasing Curve Not Approaching Zero			Stable Curve at Zero for Approximately 15 Days			Rising Curve		
	IRR	95% CI	*p* Value	IRR	95% CI	*p* Value	IRR	95% CI	*p* Value
Veneto region	1.14	1.04, 1.24	0.001	3.07	2.21, 4.29	<0.001	1.07	1.03, 1.11	<0.001
Mod1 vs. Mod2	1.41	0.79, 2.56	0.2	0.60	0.03, 12.40	0.7	1.04	0.86, 1.25	0.7
Mod3 vs. Mod2	1.29	0.85, 1.96	0.2	0.14	0.01, 1.43	0.1	1.06	0.90, 1.25	0.5
Mod4 vs. Mod2	2.00	1.00, 4.00	0.04	0.07	0.00, 4.80	0.2	1.32	1.02, 1.71	0.04

Mod1 = first organizational model, Mod2 = second organizational model, Mod3 = third organizational model, Mod4 = fourth organizational model, IRR = incidence rate ratio, CI = confidence interval.

## Data Availability

The data supporting the findings of this study are available upon reasonable request from the corresponding author.

## Supplementary Materials

The following supporting information can be downloaded at https://www.mdpi.com/article/10.3390/healthcare13030268/s1: Figure S1, Daily contact tracing (CT) activity for each organizational model in the three periods considered. The CT activity is proportional to the number of cases of each organizational model. Figure S2, Daily trend of hospitalizations for each organizational model. The number of hospitalizations is proportional to the number of COVID-19-positive cases in each organizational model.
