# Supplementary material for: Contact Tracing During the COVID-19 Epidemic: Insights from the Experience of the Veneto Region in Italy
Source: Healthcare (Basel). 2025 Jan 30;13(3):268. doi: 10.3390/healthcare13030268 (PMC11816897; doi:10.3390/healthcare13030268)

Supplemental material

Figure S1. Daily contact tracing (CT) activity for each organizational model in the three periods considered. The CT activity is proportioned to the number of cases of each organizational model.

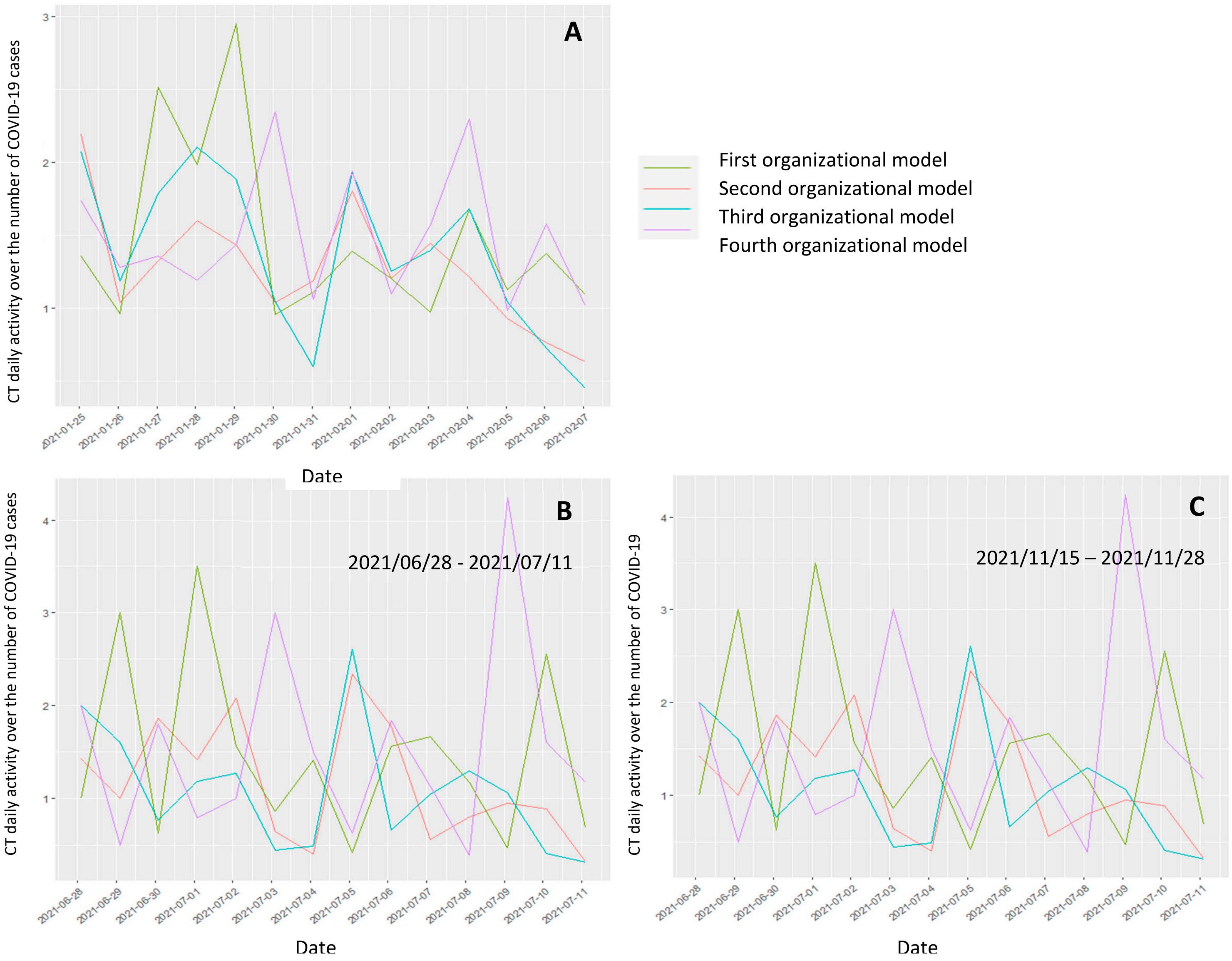

Figure S2

Daily trend of hospitalizations for each organizational model. Hospitalizations are proportioned to the number of COVID-19 positive cases of each organizational model.

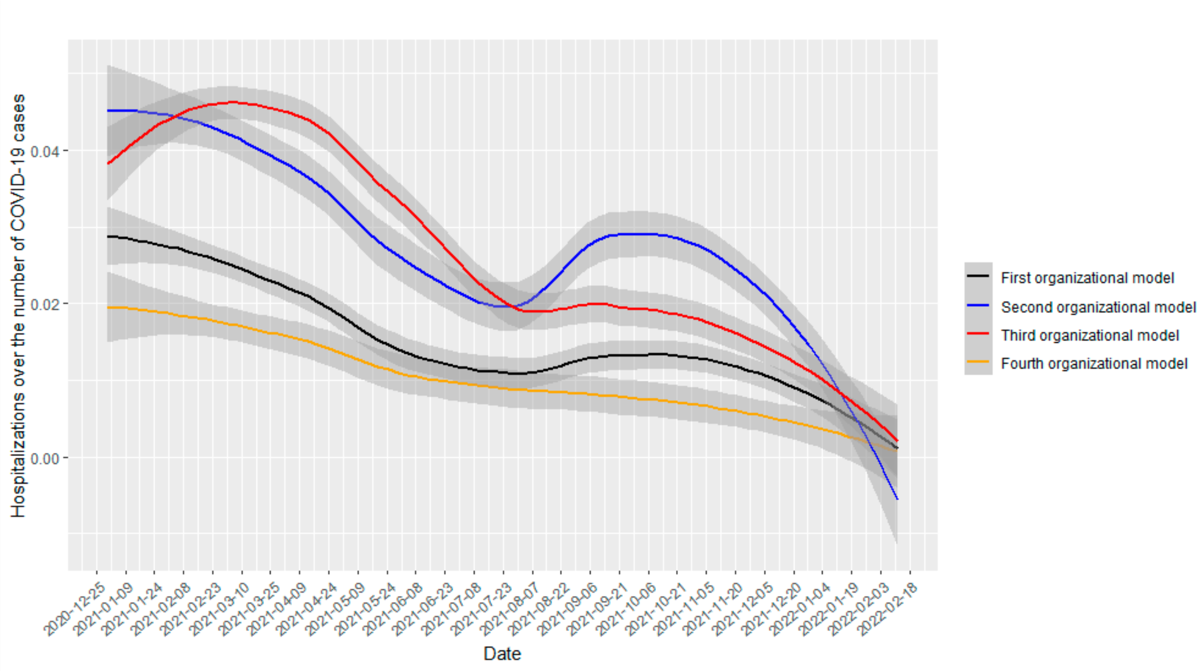

Supplement: Supplementary file 1 [file healthcare-13-00268-s001.zip › healthcare-3353169-supplementary.pdf]
